# Does opioid therapy enhance quality of life in patients suffering from chronic non-malignant pain? A systematic review and meta-analysis

**DOI:** 10.1177/20494637231216352

**Published:** 2023-11-26

**Authors:** Karl V. L. Kraft, Teresa Backmund, Leopold Eberhart, Ann-Kristin Schubert, Hanns-Christian Dinges, Maria K. Hagen, Markus Gehling

**Affiliations:** 1 Department of Anaesthesiology and Intensive Care Medicine, University Hospital Marburg, 9377 Philipps University of Marburg, Marburg, Germany; 2 Department of Physics and Material Sciences Center, 9377 Philipps-University Marburg, Marburg, Germany; 3 Pain Center Kassel, Kassel, Germany

**Keywords:** Chronic pain, health related quality of life, opioid, quality of life

## Abstract

**Background and objective:**

Chronic pain is associated with a poor health-related quality of life (HRQL). Whereas the prescription rate of opioids increased during the last decades, their use in chronic non-malignant pain remains unclear. However, there is currently no clinical consensus or evidence-based guidelines that consider the long-term effects of opioid therapy on HRQL in patients with chronic non-cancer pain. This systematic review aims to address the question of whether opioid therapy improves HRQL in patients with chronic non-malignant pain and provide some guidance to practitioners.

**Databases and data treatment:**

PubMed, EMBASE and CENTRAL were searched in June 2020 for double-blind, randomized trials (RCTs), comparing opioid therapy to placebo and assessed a HRQL questionnaire. The review comprises a qualitative vote counting approach and a meta-analysis of the Short Form Health Survey (SF-36), EQ-5D questionnaire and the pain interference scale of the Brief pain inventory (BPI).

**Results:**

35 RCTs were included, of which the majority reported a positive effect of opioids for the EQ-5D, the BPI and the physical component score (PCS) of the SF-36 compared to placebo. The meta-analysis of the PCS showed a mean difference of 1.82 [confidence interval: 1.32, 2.32], the meta-analysis of the EQ-5D proved a significant advantage of 0.06 [0.00, 0.12]. In the qualitative analysis of the mental component score (MCS) of the SF-36, no positive or negative trend was seen. No significant differences were seen in the MCS (MD: 0.65 [-0.43, 1.73]). A slightly higher premature dropout rate was found in the opioid group (risk difference: 0.04 [0.00, 0.07], *p* = .07). The body of evidence is graded as low to medium.

**Conclusion:**

Opioids have a statistically significant, but small and clinical not relevant effect on the physical dimensions of HRQL, whereas there is no effect on mental dimensions of HRQL in patients with chronic non-malignant pain during the initial months of treatment. In clinical practice, opioid prescriptions for chronic non-cancer pain should be individually assessed as their broad efficacy in improving quality of life is not confirmed. The duration of opioid treatment should be determined carefully, as this review primarily focuses on the initial months of therapy.

## Introduction

Chronic pain affects an estimated 10–30% of the world population^1–3^ and is a major health issue with high social and economic impact.^4,5^ Chronic pain, which is usually defined as lasting for at least three to six months,^6^ affects the patient’s sleep,^7^ mood,^8,9^ social life^4,10^; finally it deteriorates the overall quality of life.^11–14^

Opioids are among the oldest and most powerful agents and are widely used in the treatment of chronic pain. The analgesic potential and efficacy of opioids is associated with various adverse effects, ranging from constipation and emesis to dependency and overdose.^15–17^ Nevertheless, the worldwide number of opioid prescriptions has increased significantly over the last decades.^18–20^ In 2016, 4% of the adult population in the US misused prescription opioids, while 33,000 people died due to opioid overdose.^21,22^ The US administration declared in 2017 the opioid crisis a public health emergency. The opioid crisis in the US has led to discussion to what extent medical prescription practices need to be reconsidered.^23^

Whereas opioids are accepted as treatment for acute severe^24^ or malignant pain,^25^ their use in chronic, non-malignant pain remains controversial.^26–28^ Although an average reduction in pain by approximately one-third can be anticipated with opioid therapy for chronic non-malignant pain in the initial months of treatment, it is imperative to consider that the adverse effects, including dependency and overdosing, increase with dosage.^29^ Given that chronic pain detrimentally impacts various dimensions of quality of life, successful therapy should also strive to effectively ameliorate these facets.

The concept of health related quality of life (HRQL) focuses on pertinent aspects of mental and physical health within the broader framework of quality of life.^30^ Still, there is no clinical consensus and evidence-based guidelines taking the long-term effect of opioid therapy (> 12 weeks) on HRQL into account in patients suffering chronical non-cancer pain.^28,31,32^

Two systematic reviews were conducted to assess the long-term effects of opioids in chronic pain, with HRQL as one of the endpoints analyzed. Chou et al. included no suitable study to examine HRQL,^33^ whereas Noble et al. determined the findings to be inconclusive due to the heterogeneity observed across the included studies.^34^ Another review focussing on opioids in neuropathic pain considered HRQL as a secondary endpoint, but could not demonstrate an improvement.^35^ Devulder et al.^36^ conducted a review that examined the impact of opioids on HRQL in chronic non-malignant pain. The review encompassed eleven studies and revealed low to medium quality evidence suggesting an improvement in HRQL as a result of opioid therapy. In a more recent analysis, a total of 19 studies were included, out of which eleven were double-blind placebo-controlled randomized controlled trials (RCT´s). Their statistical analysis incorporated data from five placebo-controlled RCT´s, along with data from studies comparing different opioid interventions. The findings of this study provided evidence supporting the improvement of physical HRQL with opioid therapy.^37^

These findings of literature research warrant reevaluation, as there is potential for expanding the methodological analysis beyond previous analyses. Additionally, conducting an updated literature search holds the potential to yield more precise and comprehensive insights into the effects of opioid therapy on HRQL in patients with chronic non-malignant pain.

## Methods

### Study registration and reporting

The present study was prospectively registered at PROSPERO (CRD42017073979) on 16 August 2017. The reporting followed the Preferred Reporting Items for Systematic Reviews and Meta-analyses (PRISMA statement).^38^

### Search strategy and data sources

To systematically frame the clinical question regarding the impact of opioids on HRQL in chronic non-malignant pain and to align it with a scientific format, the patient, intervention, control, outcome, study (PICOS) scheme^39^ was employed. The search strategy was implemented in the following manner: P (patients) patients suffering from chronic non-malignant pain, I (intervention) opioid therapy, C (comparison): placebo, O (outcome): quality of life assessed with a generic HRQL measurement as primary or secondary outcome, S (study design): randomized controlled trials. The PICOS scheme^39^ of this review is depicted in Table 1.

**Table 1.** PICOS scheme of this review.

Patient	Patients suffering from chronic non-malignant pain
**I**ntervention	Opioid therapy
**C**omparison	Placebo
**O**utcome	HRQL
**S**tudy design	Randomized controlled trials

The research strategy utilized three primary keywords: chronic pain, opioids and quality of life. These keywords were combined using the operator ‘AND’ to form a logical conjunction. Additionally, synonyms for each keyword (i.e. long-term pain and persistent pain) were included using the operator ‘OR’, forming a logical disjunction. Study search was further restricted to studies published in English, French, German, Spanish and Italian language.

In June 2020, a comprehensive search was conducted across the following databases: PubMed, EMBASE and The Cochrane Library.

### Inclusion and exclusion criteria

In this analysis, studies were eligible for inclusion, if they focused on adult patients (18 years and above) with chronic non-malignant pain and employed a randomized placebo-controlled design. Furthermore, studies had to asses HRQL with a generic assessment tool, like SF-36^40^ or EQ-5D,^41^ either as a primary or secondary outcome measure to be included in the meta-analysis. The SF-36 consists of eight dimensions which can be collapsed into two global summary scores, the Physical Component Summary (PCS), and Mental Component Summary (MCS) referring to mental and physical dimensions of HRQL.^40^ The EQ-5D also allows for converting the single dimensions into a single summary index.^41^ Questionnaires that examine the interference of pain with daily living-like the Pain Interference Scale of the Brief Pain Inventory (BPI)^42^- were evaluated in the qualitative, vote counting, part of this analysis. The BPI serves as a complement tool investigating the general impact of pain on daily living.

We did not impose any restrictions based on the type of pain (nociceptive, neuropathic or mixed). The kind of opioid administration had to be non-invasive, such as oral or transdermal. Exclusion criteria encompassed the minority of patients included in the study (under 18 years old), chronic malignant pain and invasive administration of opioids, such as intrathecal opioid administration. Studies assessing exclusively disease-specific HRQL or functionality measures, that is, the WOMAC scale in the case of osteoarthritis, were not included in this review. Furthermore, comparative studies involving different opioids without a placebo control were excluded. Regarding the duration of the double-blind period or the length of follow-up, no restrictions were made.

### Data extraction

All search results were imported into a reference management software and duplicates were removed. Title and abstract of studies were screened, and full text of studies, potentially to be included, was retrieved. Full text papers were checked against the inclusion and exclusion criteria again. At this point, reasons for exclusion of any study were documented. Screening of studies and the determination of study inclusion were conducted by two independent authors. Discrepancies were resolved by consensual agreement. Data of the included studies were extracted using a standardized form and subsequently transferred into a spreadsheet. Extracted data includes information on study type, duration, number of patients enrolled and included in efficacy analysis, inclusion and exclusion criteria, interventions, HRQL measures, additional assessments and funding. For missing data, corresponding authors were contacted twice.

### Risk of bias assessment

**Figure 1.1.**
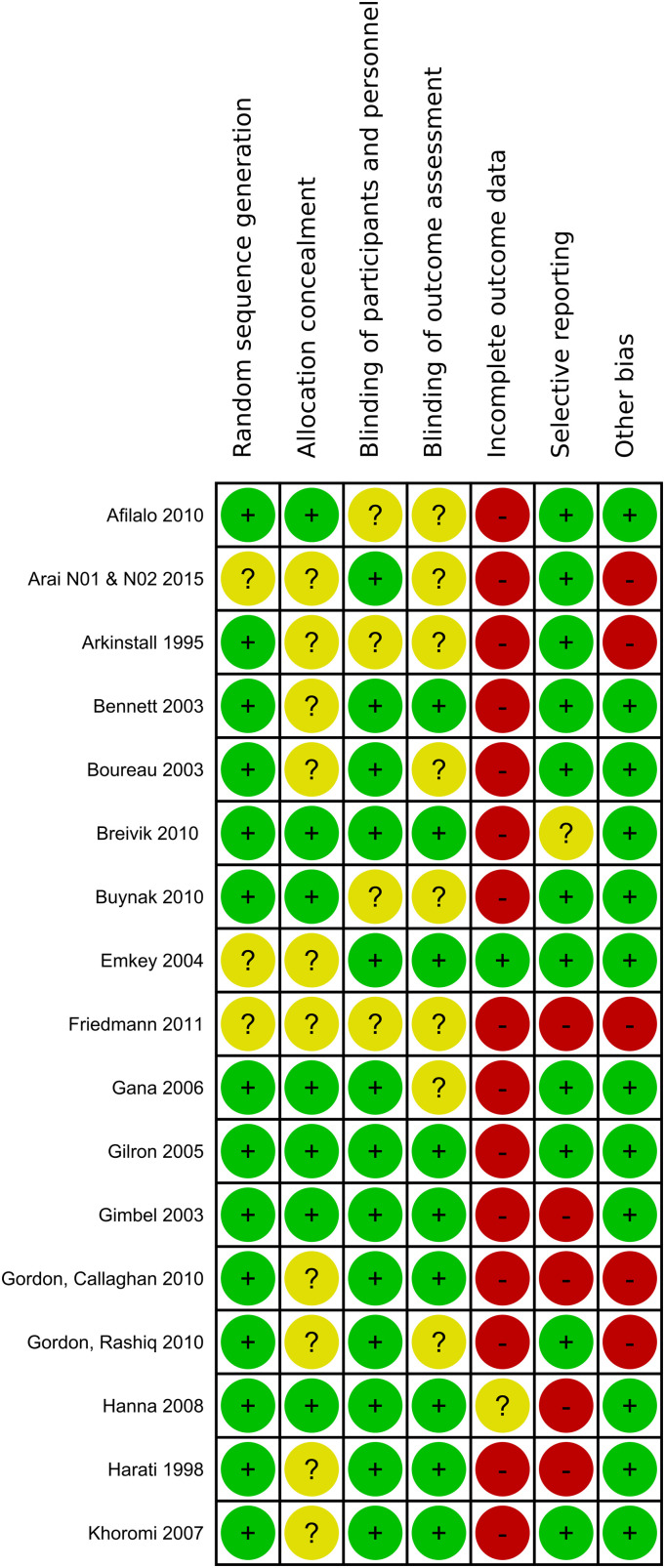
Summary of the risk of bias assessment of included studies: lalo 2010 - Khoromi.

**Figure 1.2.**
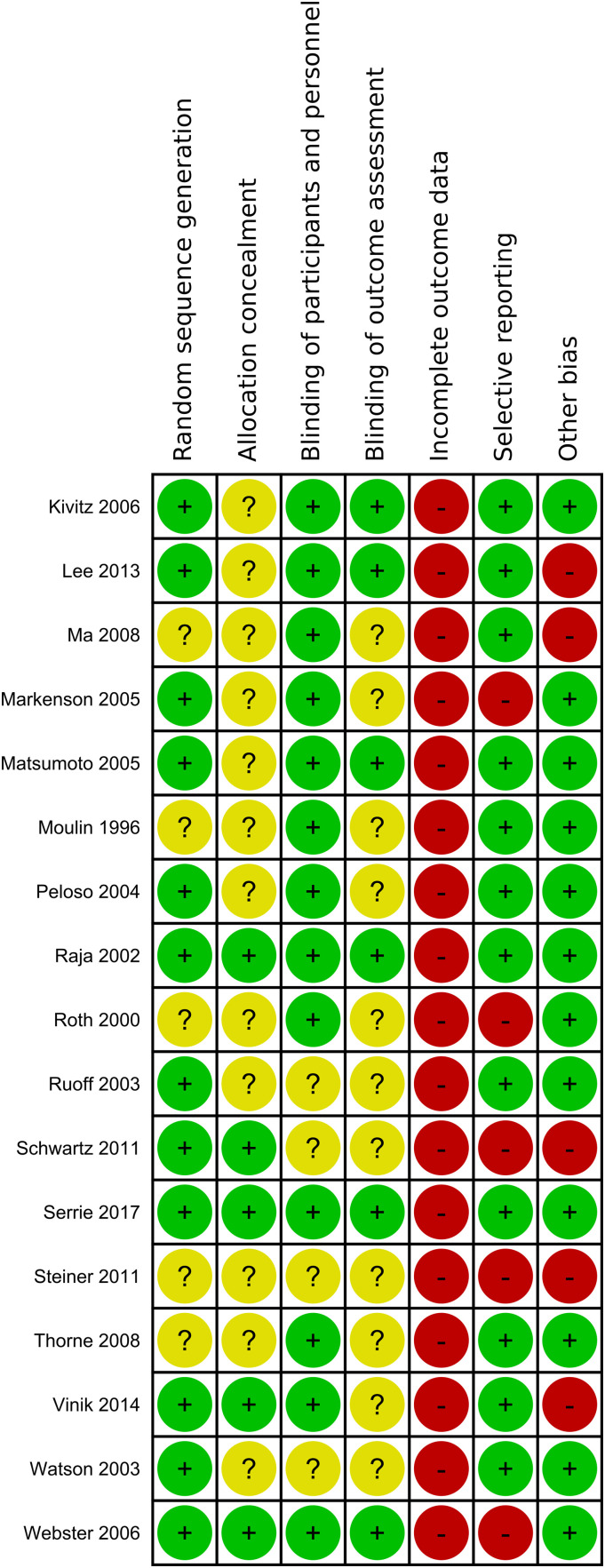
Summary of the risk of bias assessment of included studies: Kivitz 2006 - Webster.

The Cochrane Risk of Bias Tool was used for grading the methodological quality of the studies.^43^ The included studies were assessed for potential bias based on following grouped risk of bias items: randomization (random sequence generation, allocation concealment), blinding (blinding of participants and care personnel, blinding of outcome assessment and investigators), incomplete outcome data and selective reporting. One last domain describes any other potential sources of bias (Figure 1.1, 1.2).

### Vote counting

Several studies with different HRQL approaches were expected to be unsuitable for meta-analysis, that is, due to incomplete reporting of results. The vote counting approach allows for systematic comparability in the case of heterogeneous studies or incompletely reported results. In the vote counting approach only the sign of the difference between opioid and placebo is counted, that is, how many studies show a beneficial positive direction or a harmful negative direction of effect. As this refers to a numerical difference, it is not relevant if the effect is statistically significant.

Studies in which multiple opioid groups demonstrated conflicting directions of effects, without the possibility of summation, were classified as neutral. The vote counting is presented graphically according to a modified figure by Boon et al.^44^

### Statistical analysis

To conduct the quantitative meta-analysis, datasets of the physical component score (PCS) and mental component score (MCS) of the SF-36 summary scales, as well as the EQ-5D Health scale, were imported into the R software.^45^ Analysis was performed using the packages ‘meta’^46^ and ‘metafor’.^47^ In the case of continuous outcome parameters, a random-effects generic inverse variance method was employed for pooling effects. The pooled mean difference and a prediction interval were calculated. In the case of binary outcome, like the analyses of premature dropouts, Mantel-Haenszel method with a random effect model was used. Here, the effect measure was the pooled risk difference. The significance level was set to *α* = 0.05. To prevent the unit-of-analysis error, studies with multiple treatment groups were combined by pooling the different treatment groups using opioids into a single group. This combined group was then compared to the placebo treatment group. Finally, some studies only reported the single dimensions of the SF-36 questionnaire and did not calculate the summary scales PCS and MCS. In this case, the summary scales were calculated and the correlation matrix was computed according to Ware’s SF-36 Manual.^40^ Heterogeneity was tested with the Chi^2^ and I^2^-Test. We assessed the distribution of effects and explored the potential presence of publication bias using funnel plots. Asymmetry of funnel plots was statistically analyzed by Egger’s test.^48^ In case of a significant asymmetry, the trim and fill method^49^ estimates a corrected pooled mean difference.^50^

Standard deviation of mean change was lacking in some studies. To estimate these values, a correlation coefficient of 0.6 was calculated between baseline and post-treatment measurements, using data from Peloso 2004^51^ and Ruoff 2003.^52^

However, due to variations in study design and the statistical estimation of standard deviation of mean change, a sensitivity analysis was conducted. This analysis involved excluding studies that utilized an enriched or withdrawal design, as well as other designs that favoured the active treatment group.

To determine the clinical significance or relevance of a significant result, the minimal clinically important difference (MID) was conducted. The model of the MID can serve as a valid starting pointpoint.^53–55^

## Results

### Literature search

**Figure 2.**
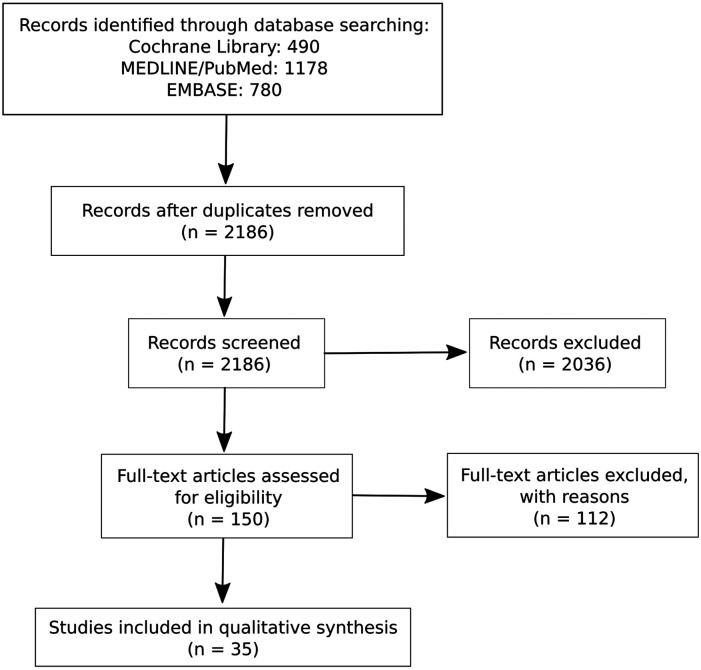
Flowchart of literature search.

After removal of duplicates, a total of 2186 references were screened, and 150 full-text articles were assessed (Figure 2). Finally, 38 full-text articles were included in the analyses, representing 35 different studies.

### Study characteristics

Out of 35 studies included, 11 reports dealt with chronic pain due to osteoarthritis, eight described chronic low back pain, and 11 studies investigated patients suffering from neuropathic pain. Four studies included patients with other conditions (i.e. fibromyalgia).

### Risk of bias

**Figure 3.**
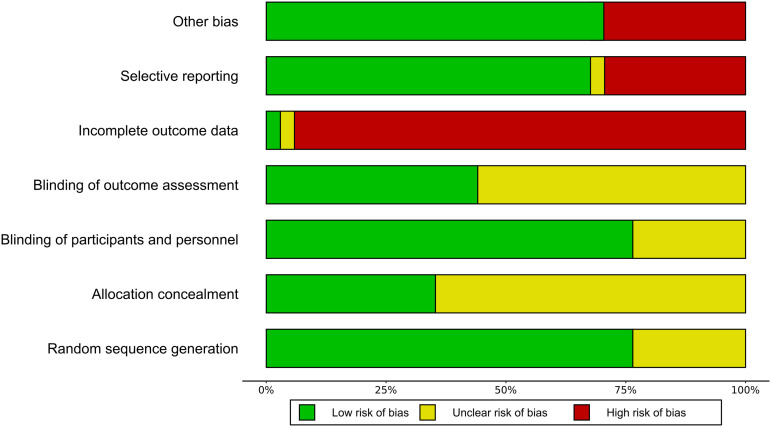
Risk of Bias of all studies included.

Figure 3 shows the Risk-of-Bias summary of all studies. The risk of bias was found to be high in the dimension of incomplete outcome data, indicating a significant presence of attrition bias. This was primarily attributed to a significant rate of premature dropouts, surpassing the cut-off value for high risk with 30% of study participants for studies lasting at least 12 weeks or 20% for studies with a shorter duration. High-risk bias was also observed due to the implementation of an enriched enrolment strategy, which may lead to an overestimation of treatment effect or an underestimation of adverse events.

### Qualitative synthesis

In total, out of the 26 studies that assessed the PCS scale of the SF-36, 22 studies showed a positive direction of effects, indicating a beneficial effect of the opioid therapy. Three studies reported no effect or did not provide sufficient information, while one study demonstrated a negative effect. In the vote counting analysis of the 25 studies assessing the MCS scale, ten studies exhibited a positive trend, while nine studies demonstrated a negative result. Six studies were classified as neutral. Among the seven studies that reported the EQ-5D Health scale, four studies indicated a positive effect, two studies reported no effect or sufficient information, and one study showed a negative effect of the opioid therapy compared to placebo. Six of seven studies reporting the BPI pain interference scale showed a positive result, while one study did not report sufficient information. The Tables 2, 3 and 4 show the vote counting, grouped by the underlying pain conditions osteoarthritis, low back pain and neuropathic pain.

**Table 2.** Effect directions in studies assessing osteoarthritis pain. The scores depicted are the SF-36. The scores depicted are the SF-36 PCS and MCS summury scales, the EQ-5D Health scale, the BPI pain interference scale and other HRQL measures. Studies in which there are multiple opioid groups showing positive and negatice effect directions were considered neutral. The signs differ in size, regarding the number of patients assessed for HRQL in the study. ▲: positive effect of opioid, ◂▸: no change/neutral/mixed effects/no sufficient information, ▼: negative effect of opioid. Tap: tapendadol, Oxc: oxycodone, Oxm: oxymorphone, Tra/APAP: tramadol/acetaminophen, Tra: tramadol, Bup: buprenorphine. Sample size: ▲: 0-200, ▲: 201-500, ▲: > 500.

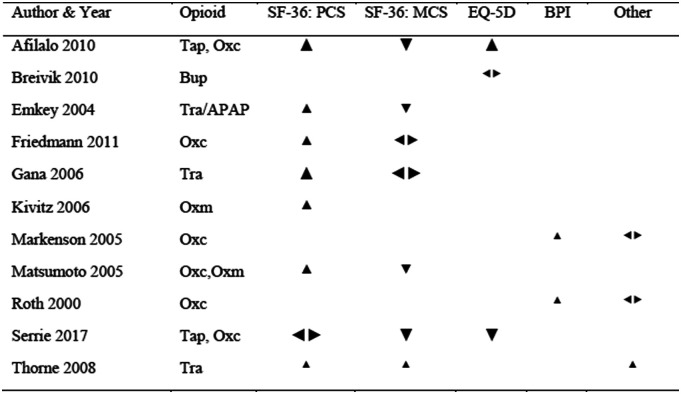

**Table 3.** Effect directions in studies assessing low back pain. ▲: positive effect of opioid, ◂▸: no change/neutral/mixed effects/no sufficient information, ▼: negative effect of opioid. Tap: tapentadol, Oxc: oxycodone, Oxm: oxymorphone, Tra/APAP: tramadol/acetaminophen, Tra: tramadol, Bup: buprenorphine. Sample size: ▲: 0-200, ▲: 201-500, ▲: > 500.

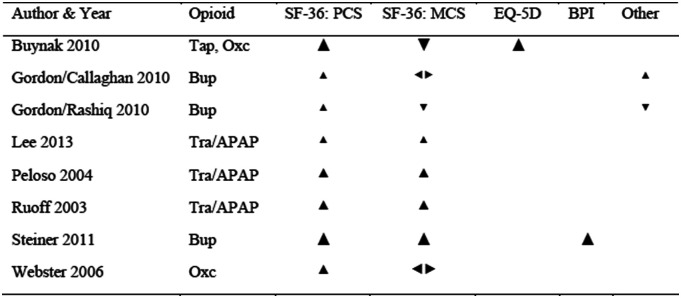

**Table 4.** Effect directions in studies assessing neuropathic pain. ▲: positive effect of opioid, ◂▸: no change/mixed effects/no sufficient information, ▼: negative effect of opioid. Tap: tapentadol, Oxc: oxycodone, Fen: fentanyl, Tra: tramadol, Mor: morphine, Met: methadone. Sample size: ▲: 0-200, ▲: 201-500, ▲: > 500. * The results of Schwartz 2011 are only reported in a pooled analysis and are therefore regarded as neutral.

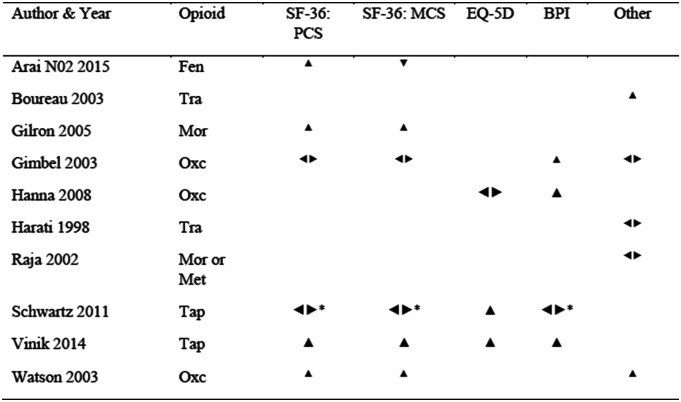

### Meta-Analysis

Among the 26 studies included in the vote counting analysis of the SF-36 and SF-12 scales, a total of 18 studies provided data that were suitable for meta-analysis. One study reported only data of the PCS.^56^ Five of seven studies included in the EQ-5D vote counting were suitable for meta-analysis.

#### SF-36 PCS

**Figure 4.**
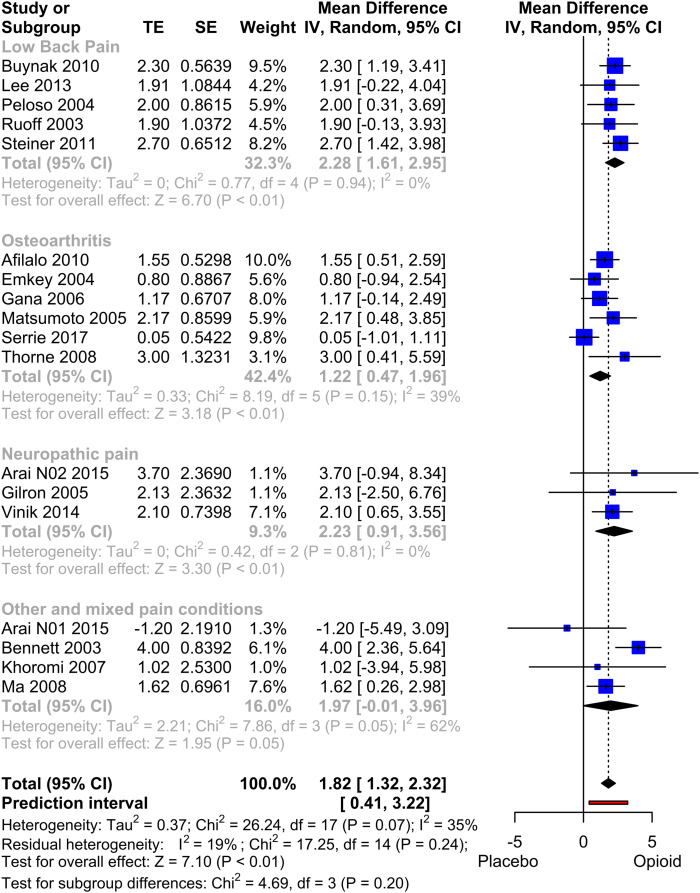
Forest Plot of PCS (physical quality of life). Meta-analysis of all PCS datasets. Mean differences between Opioid and Placebo. A generic inverse variance method with random effects was used. Pooled estimates of the weighted mean difference are shown with 95% CIs. Pooled effect estimates are presented as diamonds and lines depict the 95% CIs.

The meta-analysis of the PCS of the SF-36 scale comprised 18 studies, involving a total of 7391 patients (Figure 4). The overall effect size, calculated as the mean difference, demonstrated a statistically significant advantage in favour of the opioid group (mean difference: 1.82, *p* < .001). A significant advantage within this range was also observed in the three subgroups Low Back Pain, Osteoarthritis and Neuropathic Pain. Despite the presence of moderate heterogeneity (I^2^ = 35%, indicating some degree of variability in the results), the prediction interval ranging from 0.41 to 3.22 provided further confirmation of the beneficial effect. A funnel plot of the studies included showed a symmetrical distribution with no clear evidence of publication bias. A sensitivity analysis was conducted, excluding studies that employed an enriched or withdrawal design^57–59^ or other designs favouring the active treatment group.^60^ The results of this analysis showed a slightly lower mean difference of 1.75 (*p* < .001) in favour of opioid. Further exclusion of studies in which the PCS was estimated^61–63^ resulted in a mean difference of 1.77 (*p* < .001) with an increase in heterogeneity (I^2^ = 57%).

However, our analyses using different approaches for the MID, such as taking the standard deviation of the baseline value^54^ or employing other published approaches,^55^ did not demonstrate clinical significance for the observed advantage in physical HRQL.

#### SF-36 MCS

**Figure 5.**
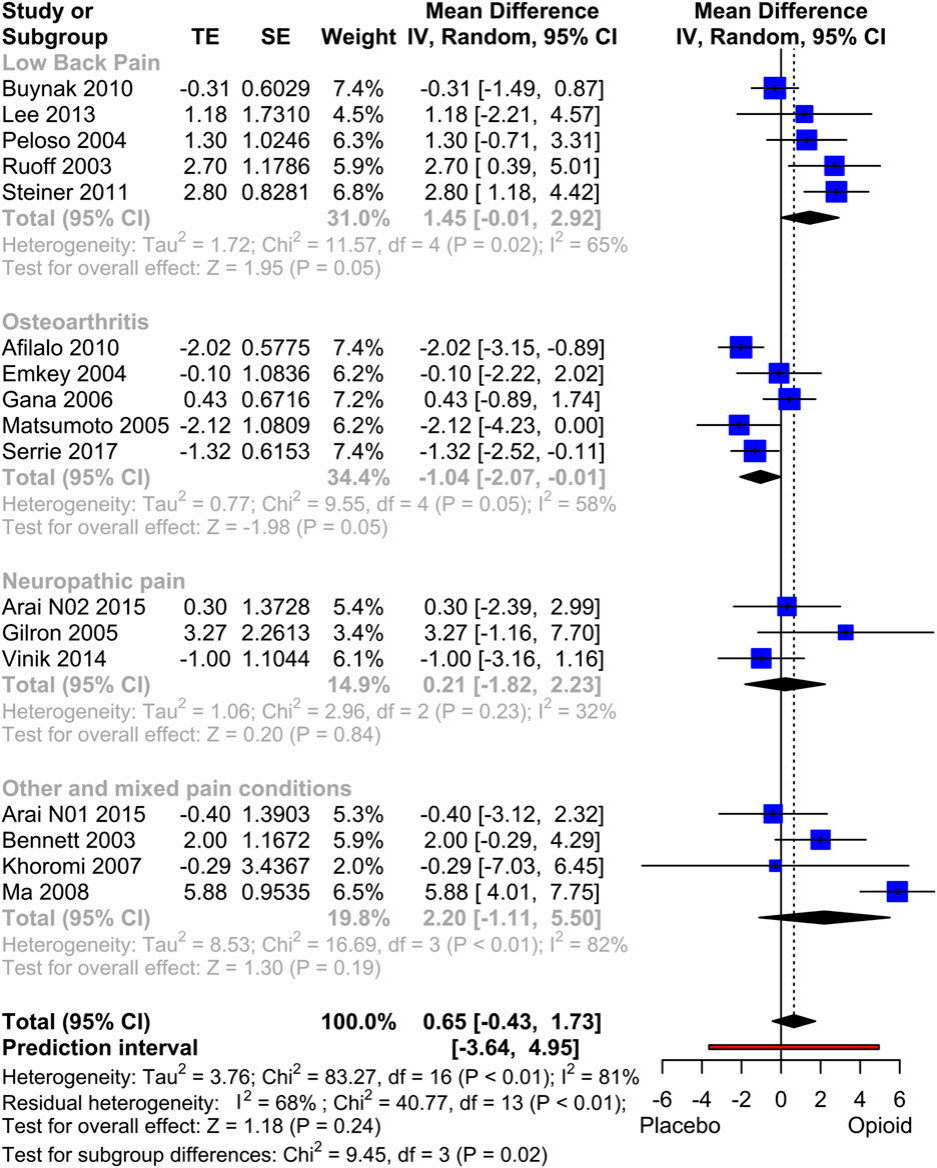
Forest Plot of MCS (physical quality of life). Meta-analysis of all MCS datasets. Mean differences between Opioid and Placebo. A generic inverse variance method with random effects was used. Pooled estimates of the weighted mean difference are shown with 95% CIs. Pooled effect estimates are presented as diamonds and lines depict the 95% CIs.

17 studies, comprising data from 7237 patients, were included for quantitative analysis of MCS. The analysis (Figure 5) did not reveal significant outcome differences between opioid and placebo (MD: 0.65, *p* = .24). The subgroup analysis of Osteoarthritis demonstrated a significant worsening of the opioid therapy compared to placebo (*p* = .048), while the Low Back Pain subgroup showed a trend towards an advantage in the opioid group, although it did not reach statistical significance (*p* = .052). In a sensitivity analysis, excluding studies that used study designs likely to favour the opioid treatment,^57–60^ the overall effect shifted towards zero, resulting in a mean difference of 0.07 (*p* = .88). Other sensitivity analyses did not reveal any statistically significant differences. The funnel plot exhibited a slight visual asymmetry, but subsequent testing using Egger's test did not indicate a significant asymmetry (*p* = .18).

An additional subgroup analysis of MCS and PCS demonstrated no statistically significant differences between studies with a duration less than 12 weeks and studies with longer durations. A preliminary analysis using Spearman correlation also showed no significant correlation between effect size and study duration.

#### EQ-5D

**Figure 6.**
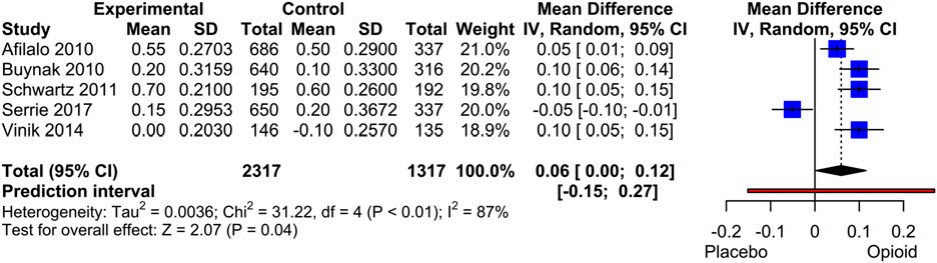
Forest Plot of EQ-5D. Mean differences between Opioid and Placebo. A generic inverse variance method with random effects was used. Pooled estimates of the weighted mean difference are shown with 95% CIs. Pooled effect estimates are presented as diamonds and lines depict the 95% CIs.

The meta-analysis included five studies with a total of 3634 patients. The results showed a significant mean difference of 0.06 (*p* = .045) in favour of the opioid treatment (Figure 6). However, a sensitivity analysis that excluded studies with withdrawal or enriched design^59,64^ demonstrated no significant treatment effect (MD: 0.03, *p* = .43). Due to the inclusion of fewer than 10 studies, a funnel plot was not conducted.

### Premature study withdrawals

**Figure 7.**
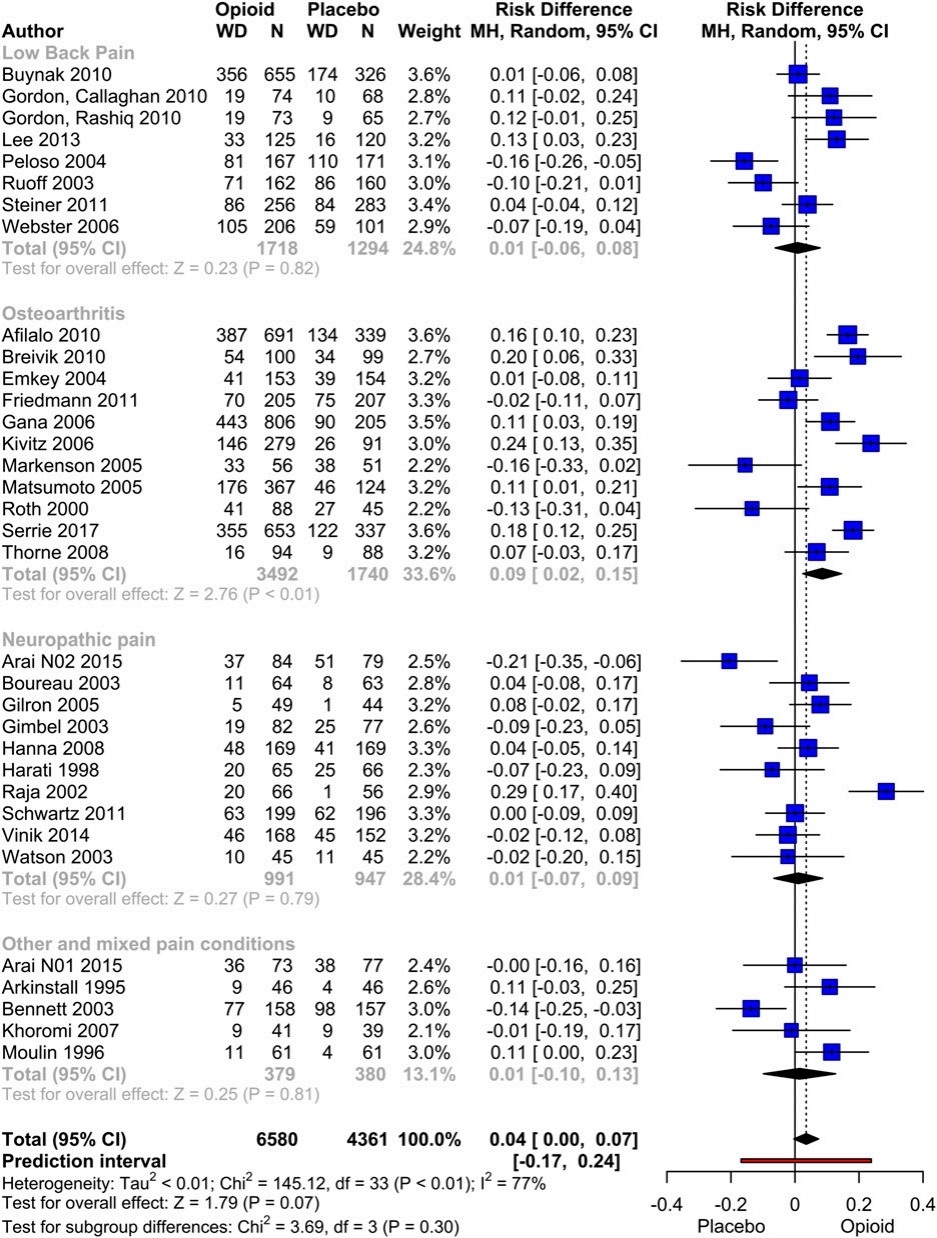
Meta-analysis of premature withdrawals. Pooled Risk Difference of Opioid compared to Placebo. Mantel-Haenszel method with a random effect model was used. Pooled effect estimates are presented as diamonds and lines depict the 95% CIs.

A meta-analysis of study withdrawals was conducted, including a total of 34 pooled studies with data from 6580 patients (Figure 7). Overall, the opioid group exhibited a slightly higher risk of premature withdrawal, although the difference did not reach statistical significance (risk difference mean: 0.04, *p* = .07). However, in the subgroup analysis of studies evaluating osteoarthritis pain, the opioid group demonstrated a significantly higher rate of withdrawals compared to the placebo group (RD: 0.09, *p* = .006).

The meta-analysis of study withdrawals due to adverse events (AE) was performed on 28 studies which provided data of 6183 patients. The risk of withdrawals due to AE was significantly higher in the opioid group compared to the placebo group (RD: 0.15, *p* < .001). The subgroup analyses based on the underlying diseases showed consistent findings with regards to withdrawals. Sensitivity analyses conducted did not significantly alter the results.

A total of 27 studies, providing data of 6183 patients, were included in the quantitative analysis of withdrawals due to lack of therapeutical efficacy. The analysis revealed a significant higher risk of withdrawal in the placebo group due to lack of efficacy (RD: −0.11, *p* < .001). This effect remained significant across all subgroups analyzed. The funnel plot of the effects exhibited a considerable heterogeneity, which was further supported by the results of the Eggers test (*p* < .001), suggesting a potential overestimation of the effect due to bias. By employing Duval´s trim-and-fill procedure, a statistical estimation of the true risk difference yielded a corrected value of −0.05 (*p* = .010). This estimation suggests that the actual effect size may be smaller than what was observed in the original meta-analysis incorporation all available data.

## Discussion

This review studies the impact of opioids on the HRQL of patients suffering from non-cancer chronic pain. It comprises data of 35 different RCTs, representing 11,057 patients. Only placebo-controlled RCTs were included, which provide the highest grade of evidence. A comprehensive search strategy was chosen to cover as many different types of chronic non-malignant pain and opioids as possible and to get a quite extensive overview over the state of evidence.

The meta-analysis of HRQL in this review focuses on this well-defined summary indices as they facilitate the statistical analysis of HRQL and provide a global model of clinically relevant HRQL. Our primary focus was on outcomes from HRQL studies using SF-36^40^ and the EQ-5D.^41^ Additionally, our qualitative vote counting approach evaluated other general HRQL measurements and the Pain Interference Scale of the Brief Pain Inventory (BPI) (Charles, S. and Cleeland, P.). While our main emphasis was on general insights into chronic non-cancer pain and HRQL, disease-specific questionnaires were intentionally excluded to maintain this broader perspective.

The meta-analysis showed a small benefit of the opioid therapy over placebo indicated by physical HRQL. Despite the demonstrated statistical significance of the benefit of opioid therapy on physical HRQL, the authors consider the clinical relevance of this effect, as determined by the MID, to be minor. In contrast to the physical HRQL, the mental HRQL showed no significant difference between opioid and placebo. In sensitivity analyses the direction of effect changed to a negative direction.

Since not all studies report the information necessary for a statistical meta-analysis, this review consists of an additive qualitative analysis, which is structured by a vote counting approach. In the qualitative vote counting approach most studies showed a benefit for opioid therapy in the PCS scale and the EQ-5D index. In the vote counting analysis of the MCS scale, there was a nearly equal distribution between studies indicating a negative effect and those showing positive effects.

Thus, although chronic pain is generally associated with deterioration of mental health,^65^ no effect of opioid therapy in the mental dimensions of HRQL could be demonstrated. A possible explanation might be that pain reduction in general does not show any strong effect on the mental HRQL dimension, or that an improvement in mental health might only show up in the case of a longer treatment duration. However, there is evidence that the severity of pain correlates with quality of life in the physical and mental domains.^66–68^ Another likely reason may be that common mental or neurological side effects of opioid therapy such as dizziness or somnolence neutralize a possible beneficial effect on mental HRQL. One possible rationale is the activation of kappa receptors by opioids, which, unlike the activation of mu and delta receptors, can lead to dysphoria. This adverse effect of opioid therapy could contribute to the development of opioid-associated depression.^69,70^ Moreover, several studies have demonstrated a significant association between opioid dose and the incidence of depression.^69,71,72^ Through the evaluation of the longitudinal trajectory, some studies identified that the connections between opioid therapy and the incidence of (new-onset or recurrent) depression were not influenced by pain or other factors that would potentially have a causal role.^73–75^

The analysis of premature withdrawals also confirms the importance of side effects for therapeutical adherence. While patients in the placebo group dropped out mainly due to lack of efficacy, during opioid therapy patients dropped out significantly more often due to adverse events. The overall risk for premature withdrawal was higher in the opioid group than in the placebo group, however, not showing statistical significance in the main analysis but in sensitivity analyses. This is also confirmed by an earlier meta-analysis of studies investigating osteoarthritis pain, in which a significant higher withdrawal rate under opioid therapy caused by adverse events was demonstrated.^76^ It seems that for many participants, the side effects of opioid therapy were outweighing the positive effect on pain relief and HRQL, leading to dissatisfaction with therapy.

Due to the high overall withdrawal rate of approximately 41% of the participants, we found a high risk of attrition bias. A high dropout rate lowers the external and internal validity of the study, as the study population at the endpoint may not represent the study population at randomization or the general population suffering from chronic pain. Other frequent factors that limited the validity of the studies and resulted in a substantial risk of bias were incomplete reporting of non-significant HRQL results on the one hand, or the use of an ‘enriched’ study design on the other hand. Both factors potentially result in an overestimation of the treatment effect. Study designs, such as the ‘enriched design’, preselect the study sample by initially administering the opioid medication to all patients in an open-label phase and later only randomize patients who tolerate the medication well or who show a beneficial treatment response. In general, using an enriched design may bear the risk of unblinding due to side effects, as all participants experience possible side effects during the initial open-label phase.^77^ There is also a tendency to underestimate the number of adverse events.^78,79^ Other authors argue that studies using enriched design may have a higher potential to detect small effects in subgroups and separation into responders and non-responders resembles clinical practice.^80,81^ However, as this review addresses the effect of opioids on HRQL in general, we think that an ‘enriched’ study design may decrease internal and external validity. Therefore, sensitivity analyses were used to detect a bias on the effect measures. Although we found no significant differences, the analysis showed a tendency to larger positive effects, when studies with enriched design were included.

The study population is predominantly female (61.3%) and has a mean age of 57.3 years, reflecting the distribution of chronic pain in the general population.^82^ An important difference to clinical reality is that multi-morbid patients are mostly excluded from studies, although multi-morbidity is clearly associated with chronic pain.^83,84^ This may limit the external validity of this review.

The studies included in our analysis show a high clinical heterogeneity, covering a broad spectrum of different diseases and opioids. Most frequently, studies on chronic low back pain, osteoarthritis or neuropathic diseases were found. Neuropathic pain was mainly caused by post-herpetic neuralgia and diabetic polyneuropathy. Other studies also dealt with fibromyalgia or neck pain. Overall, these conditions account for a large proportion of chronic pain conditions in the general population.^85^ Studies on other frequent pain conditions like trigeminus neuralgia or migraine were not found, as opioids are generally not recommended as main therapy in these specific cases.^83,86^ Results from this meta-analysis may not apply to these diseases. Our data provides support for the necessity of improving the stratification of indication for opioid therapy. The presence of chronic non-cancer pain alone did not identify treatment responder in a satisfactory manner.

The opioids used in the studies range from weak opioids such as tramadol and codeine to strong opioids such as fentanyl or tapentadol in different formulations. We also included trials with tramadol/acetaminophen in a fixed combination, as acetaminophen was widely used as a rescue medication in RCTs. However, the documented effects on HRQL may also depend on the individual opioid substances, which bear different pharmacological characteristics. Differences between distinct drugs or dosage regimen could be addressed by further subgroup or network analysis.

In total, 18 studies lasted at least 12 weeks, with a median study duration of 9.3 weeks. Thus, the conclusions of this analysis are primarily valid for the first months of therapy. Whether the effect of opioid on HRQL is stable over long-term period or if, for example, late side effects affect the outcome^15,87^ needs to be studied in further research works. Especially with regard to the reduction of HRQL caused by depression, the incidence of which is correlated with the duration of opioid therapy,^70,74,88^ it is important to explicitly consider this factor in future research. This question is particularly important because chronic non-cancer pain with a middle age of onset requires long-term strategies and sometimes life-time treatment.

However, because of the high clinical heterogeneity of the studies included and varying definitions of the MID, the assessment can only be a rough estimate and needs further review. Our analysis included a large number of patients. Therefore, the precision of the overall effect seems to be reliable. Regarding the magnitude of the effect, there may be subgroups of patients, which benefit more from opioid therapy, but require a more specific and selective analysis to be identified. Our subgroup analysis with respect to different diseases, pain types or opioid medication showed no significant intergroup differences.

Overall, we found a small but significant improvement of physical quality of life, but no significant effect on mental health parameters. As a consequence of the small HRQL advantage and the unwanted side effects in clinical practice, the patients who may benefit from opioid therapy should be carefully identified and treatment should be monitored closely, as suggested by German guidelines of opioid therapy in non-cancer pain.^89^ With respect to the small effect on HRQL our data raise doubts on a presumed general recommendation of opioids in chronic non-cancer pain. Chronic non-cancer pain was not a conclusive indicator for effective opioid therapy in terms of quality of life.

## Conclusions

This systematic review demonstrates an overall statistically significant, but presumably clinically not important effect of opioids on measures of physical quality of life in non-cancer pain. In contrast, no effects of opioids on mental dimensions of HRQL could be demonstrated. This means for clinical practice, that the indication for an opioid prescription needs individual control for clinical effects because a general recommendation of opioids in patients suffering from chronic non-cancer pain was not proved in terms of increased quality of life. The duration of treatment with opioids should be set carefully, keeping in mind that the current review provides only information about the first months of treatment.

## Appendix

### List of Abbreviations

AE: adverse events
APAP: acetaminophen
BPI: brief pain inventory
Bup: buprenorphine
EQ-5D: EuroQol-Group questionnaire
Fen: fentanyl
HRQL: Health related quality of life
MCS: mental component summary of the SF-36
MD: mean difference
MID: minimal clinically important difference
Mor: morphine
Oxc: oxycodone
Oxm: oxymorphone
PCS: physical component summary of the SF-36
RD: risk difference
SF-12/36: Short-Form (12/36) Health Survey
SI: Supplementary Information
Tap: tapentadol
Tra/APAP: tramadol/acetaminophen
Tra: tramadol
